# Effect of Gluten-Free Diet on Gut Microbiota Composition in Patients with Celiac Disease and Non-Celiac Gluten/Wheat Sensitivity

**DOI:** 10.3390/nu12061832

**Published:** 2020-06-19

**Authors:** Giacomo Caio, Lisa Lungaro, Nicola Segata, Matteo Guarino, Giorgio Zoli, Umberto Volta, Roberto De Giorgio

**Affiliations:** 1 Department of Morphology, Surgery and Experimental Medicine, St. Anna University Hospital, University of Ferrara, 44124 Cona, Italy; grnmtt@unife.it (M.G.); dgrrrt@unife.it (R.D.G.); 2 Celiac Center and Mucosal Immunology and Biology Research, Massachusetts General Hospital-Harvard Medical School, Boston, MA 02114, USA; 3 Department of Morphology, Surgery and Experimental Medicine, SS. Annunziata Hospital, University of Ferrara, 44042 Cento, Italy; lisa.lungaro@gmail.com (L.L.); g.zoli@ausl.fe.it (G.Z.); 4 Department of Integrated and Computational Cell Biology (CIBIO), University of Trento, 38122 Trento, Italy; nicola.segata@unitn.it; 5 Department of Medical and Surgical Sciences, University of Bologna, 40126 Bologna, Italy; umberto.volta@unibo.it

**Keywords:** celiac disease, gluten-free diet, gluten related disorders, gut dysbiosis, leaky gut, microbiome, microbiota, non-celiac gluten/wheat sensitivity

## Abstract

Celiac disease (CD) and non-celiac gluten/wheat sensitivity (NCG/WS) are the two most frequent conditions belonging to gluten-related disorders (GRDs). Both these diseases are triggered and worsened by gluten proteins ingestion, although other components, such as amylase/trypsin inhibitors (ATI) and fermentable oligosaccharides, disaccharides, monosaccharides and polyols (FODMAPs), seem to be involved in the NCG/WS onset. Therefore, the only effective treatment to date is the long-life adherence to a strictly gluten-free diet. Recently, increasing attention has been paid to the intestinal barrier, a dynamic system comprising various components, which regulate the delicate crosstalk between metabolic, motor, neuroendocrine and immunological functions. Among the elements characterizing the intestinal barrier, the microbiota plays a key role, modulating the gut integrity maintenance, the immune response and the inflammation process, linked to the CD and NCG/WS outbreak. This narrative review addresses the most recent findings on the gut microbiota modulation induced by the gluten-free diet (GFD) in healthy, CD and NCG/WS patients.

## 1. Introduction

Celiac disease (CD) and non-celiac gluten/wheat sensitivity (NCG/WS) are the two most common conditions among gluten-related disorders (GRDs), a broad spectrum of diseases evoked by gluten ingestion, which also includes wheat allergy (WA), dermatitis herpetiformis, gluten ataxia and other clinical phenotypes [1,2,3,4,5]. Gluten is constituted by a group of ethanol soluble proteins, i.e., prolamins and glutelins, found in grains like wheat (namely gliadins and glutenins, respectively), rye (secalins and secalinins), oat (avenins and avenalins) and barley (hordeins and hordenins). These proteins, rich in glutamine and proline residues, are resistant to human intestinal proteases digestion and provide elasticity to dough needed for leavening and shaping [6,7]. The full list of cereals that must be avoided depends on the specific GRD that affects the patient. For example, patients with CD must avoid wheat (comprising its varieties and derivatives such as Kamut, spelt, emmer and triticale) rye and barley. The consumption of oats, prohibited for several years, has been recently proved to be safe for CD patients [8]. Patients with WA should avoid only wheat and its varieties if they are sensitized to wheat lipid transfer proteins (LTP or Tri a14). Otherwise, WA patients sensitized to omega-5-gliadin (Tri a19) should also avoid rye and barley for possible cross-reaction between secalins and hordeins. WA patients with wheat-dependent exercise-induced anaphylaxis should only avoid consumption of wheat, barley and rye, four hours before and two hours after physical exercise and patients with an inhaled allergy to wheat (Baker’s asthma) can eat any cereal without restrictions. NCG/WS patients should avoid the same cereals as CD patients, but, in some cases, they seem to tolerate oat, Kamut and ancient wheat varieties. Several studies have demonstrated that gliadin can enhance the intestinal permeability via zonulin [9,10], a haptoglobin 2 precursor able to alter tight junction (TJ) molecular integrity, thus leading to a “leaky gut syndrome” in predisposed people [11]. The intestinal barrier is the largest surface in contact with the environment and is constituted not only by an anatomical barrier (i.e., enterocytes and TJs), but also by several other factors such as mucus, digestive enzymes, immune system, the gut–vascular barrier, enteric nervous system and the gut microbiota. Taking these key components together, the intestinal barrier can be viewed as an “anatomical-microbiological” unit acting in concert with secretory, absorptive, motor, neuroendocrine and immunological functions [12,13]. In this line, a fast-growing number of pathological conditions, including autoimmune diseases, food intolerances, allergies and sensitivities have been related to alterations of the intestinal barrier [14].

This narrative review will focus on the gut microbiota changes in response to a gluten-free diet (GFD) in CD and NCG/WS patients. Defining these aspects will help to shed light on the interplay between gut microbiota and intestinal barrier function, a major emerging pathogenetic paradigm involved in several conditions including GRDs.

## 2. Methods

This narrative review aims to describe the effects of GFD on the microbiota composition in healthy, CD and NCG/WS adult patients. In the preparation of this manuscript, we followed the narrative review checklist by the Academy of Nutrition and Dietetics. PubMed, EMBASE, MEDLINE and Science Direct databases were assessed by two authors in two independent literature searches. Literature researches were carried out in a stepwise fashion, based on title and abstract. Keywords, searched alone or in combination, were the following: gluten-free diet; GFD; celiac disease; CD; non-celiac gluten sensitivity; NCGS; non-celiac wheat sensitivity; NCWS; healthy patients; adults; microbiome; microbiota; gluten related disorders; gut dysbiosis; leaky gut. We excluded Mesh terms. The reference list of the collected papers was also considered to find any relevant articles. Articles were included if they met the following criteria: (1) describing the effects of GFD on the gut microbiota; (2) published in the last ten years (January 2009–December 2019); (3) written in English; (4) available full text.

Articles not fulfilling the inclusion criteria or not pertinent to GRDs were excluded. Here we report the number of results found: Gluten-free diet produced 337 results; GFD produced 82 results; gluten-free diet AND healthy produced 41 results; celiac disease produced 1457 results; CD AND celiac disease produced 378 results; non-celiac gluten sensitivity produced 7 results; NCGS produced 7 results; non-celiac wheat sensitivity produced 5 results; NCWS produced 5 results; gluten related disorders produced 107 results; gut dysbiosis produced 32 results; leaky gut produced 35 results; microbiota produced 809 results; microbiome produced 946 results; microbiome AND celiac disease produced 9 results; microbiome AND healthy patients produced 116 results; microbiome AND gluten-free diet produced 10 results; microbiome AND non-celiac gluten sensitivity produced 12 results; microbiome AND non-celiac wheat sensitivity produced 4 results; microbiota AND healthy patients produced 106 results; microbiota AND celiac disease produced 11 results; microbiota AND gluten-free diet produced 18 results; microbiota AND non-celiac gluten sensitivity produced 3 results; microbiota AND non-celiac wheat sensitivity produced 1 result; gluten free diet AND adult produced 337 results; celiac disease AND adult produced 1457 results; non-celiac wheat sensitivity AND adult produced 6 results; non-celiac gluten sensitivity AND adult produced 6 results; gluten free diet AND leaky gut produced 9 results; celiac disease AND leaky gut produced 11 results; non-celiac gluten sensitivity AND leaky gut produced 5 results; non-celiac wheat sensitivity AND leaky gut produced 2 results.

## 3. Celiac Disease

CD is a chronic, multisystemic immune-mediated condition evoked by gluten ingestion in genetically predisposed individuals with a worldwide prevalence of 1–1.5% [6]. Nowadays, the only effective therapy for CD is a life-long adherence to GFD, that improves symptoms and protects from complications [15,16,17,18]. The CD pathogenetic cascade starts from gluten ingestion eliciting zonulin overexpression, followed by TJ disassembly and increased intestinal permeability [10,19,20,21,22]. Gliadin peptides are deamidated by the tissue-transglutaminase (TG2) enzyme when they reach the intestinal *lamina propria*. This structural modification enhances the affinity of deamidated gliadin peptides for the HLA DQ2/DQ8 expressed on dendritic cells (or antigen-presenting cells, APC) [23]. The APC-mediated T cells activation, along with the IL-15 mediated innate immune response, enhances the inflammatory cascade, causing intestinal lymphocytosis, overexpression of natural killer receptors and enterocyte apoptosis [24,25]. Since the enterocyte loss is not sufficiently compensated by a rapid stem cell-derived epithelial replacement, crypt hyperplasia and villous atrophy occur. Moreover, when gliadin peptides get across the altered epithelial lining and reach the bloodstream, they enhance inflammation, thus spreading the immune response and causing extraintestinal manifestations [26,27]. The ubiquitous distribution of TG2, recognized as the main CD autoantigen, along with the increased intestinal permeability (i.e., leaky gut) makes CD a unique immunological systemic disorder favoring the onset of an autoimmune response also in other tissues and organs [6,28,29,30,31,32]. Although the CD pathogenesis is quite well understood, the genetic predisposition and gluten consumption are not enough to explain the disease onset since the HLA DQ2/DQ8 is present in about 30–40% of the general population, while CD affects 1–1.5% people worldwide [6]. Thus, it has been hypothesized that several environmental factors can trigger the onset of CD, such as infections (e.g., rotavirus, reovirus and enterovirus) [33,34]*,* use of antibiotics during infancy as well as quantity, quality and age of gluten introduction, breastfeeding, nutrition, mode of delivery and changes of gut microbiota [6,35]. Despite these aspects being addressed in several studies, it is still largely unclear who will develop the disease and, conversely, who is protected against CD [36,37,38]. The complex interplay between genetics, gluten and environment factors leading to CD onset could be explained by changes in the gut microbiota composition responsible for a defective intestinal barrier function (hence, “leaky gut dysbiosis”) [39,40].

## 4. Non-Celiac Gluten/Wheat Sensitivity

NCG/WS is a condition characterized by intestinal and extraintestinal symptoms occurring soon after the ingestion of gluten and/or other proteins present in cereal like wheat, spelt, triticale, rye, barley and their derivatives in patients in whom CD and WA have been excluded [41,42]. Clinical presentation ranges from IBS-like (e.g., bloating, abdominal pain, alternating bowel movements, frank diarrhoea or constipation) and/or upper gut symptoms (gastro-esophageal reflux-like symptoms, e.g., heartburn, as well as others more ascribable to functional dyspepsia such as post-prandial fullness, early satiety, nausea and vomiting) to extra-intestinal manifestations (headache, anxiety, depression, foggy mind, fibromyalgia-like symptoms and dermatitis/rashes) [43]. Notably, gluten/wheat-induced symptoms rapidly improve upon withdrawal of the offending cereals and relapse after re-challenge [44,45]. Although gluten has been the first suspected culprit of symptom generation in NCG/WS, other components of wheat and related cereals may play a pathogenetic role such as amylase/trypsin inhibitors (ATIs) and fermentable oligosaccharides, disaccharides, monosaccharides and polyols (FODMAPs) [3,46]. NCG/WS epidemiology ranges from 0.6% (in primary care) to 6% (in tertiary referral centers). Prevalence data are highly variable because NCG/WS diagnosis is still based on clinical criteria and, due to the absence of reliable biomarkers, it can be confirmed only by a double-blind placebo-controlled challenge. The identification of possible biomarkers is dependent on a thorough knowledge of NCG/WS pathogenesis that is still far from being completely understood. However, data so far acquired indicate a central role for innate immunity with increased expression of mucosal toll-like receptor 2 (TLR2), IL-10, granulocyte colony stimulating factor (GCSF), transforming growth factor alpha (TNF-α) and CXCL-10 chemokine from peripheral blood mononuclear cells (PBMCs) [47,48,49,50]. On the other hand, the presence of circulating anti-gliadin antibodies (AGA) in a significant subset of NCG/WS patients [44,51,52] along with increased levels of interferon (IFN)-gamma mRNA in the intestinal mucosa suggest a role for the adaptive immunity [53,54,55,56].

Likewise for CD, the “leaky gut-dysbiosis hypothesis” has been postulated in NCG/WS pathogenesis. Indeed, an impaired intestinal barrier has been demonstrated in NCG/WS patients both in vivo, by lactulose-mannitol test and zonulin assay (high zonulin serum levels) and ex vivo by analyzing TJ’s protein expression, claudine-15 and myosin light chain kinase activity on intestinal biopsies [49,57,58]. An altered barrier function with microbial translocation is witnessed by a number of key findings including elevated serum levels of soluble CD14 and lipopolysaccharide (LPS)-binding protein along with immune response to microbial components (LPS and flagellin) and their correlation with serum levels of intestinal fatty acid-binding protein 2 (FABP2) [59]. Notably, GFD leads to a normalization of these markers demonstrating a link between diet, intestinal barrier and systemic immune activation in NCG/WS patients. In this context and based on the constant interplay between the intestinal epithelial barrier, gut microbiota, foods and immune system, it is likely that any potential *noxae*, including dietary components, e.g., gluten and/or other wheat proteins such as ATIs, perturb this fine tuning and precipitate symptoms experienced by NCG/WS patients. Since the gut microbiota exert a major influential role on barrier function and immune maturation and response, the next sections have addressed the consequences related to dysbiosis, i.e., the changes occurring in the myriad of various germs populating the whole gut (mainly the distal segments) in CD and NCG/WS even when such patients are properly treated with GFD [60].

## 5. Features of Gut Microbiota

The term “gut microbiota” refers to the community of microorganisms, i.e., bacteria, viruses, archaea, eukaryotes, protozoa and their collective genome (named “microbiome”) populating the entire length of the gastrointestinal tract with a cranio-caudal concentration gradient (i.e., the highest density of bacteria being in the colon), which are approximately equal in number to the human cells [61,62,63,64,65]. Microbiome counts over three million genes, producing a vast number of metabolites, while the human genome consists of only about 23,000 genes [66]. Other microbial communities of the human body include the airways, skin and urogenital tract [63,66,67,68]. From a functional standpoint, the microbiota is in symbiotic relationships within the host, managing many key functions for life, such as energy harvesting, maintenance of gut integrity and the maturation of the immune system [69,70,71,72].

The whole gastrointestinal tract covers a calculated surface of about 250–400 m^2^ and receives more than 60 tons of food in a lifetime span, so it is one of the most important sites of exchange between host, microorganism’s population and body antigens [73,74]. Therefore, the interplay between microbiota and the host is of paramount importance for the maintenance of intestinal integrity. The main phyla composing the gut microbiota are *Bacteroidetes*, *Firmicutes, Actinobacteria* and, to a lower extent, *Proteobacteria* [75]. Dietary habits can modify the gut microbiota population and the homeostasis within the host, causing dysbiosis, a condition in which the taxa present in the microbiota are quantitatively “unbalanced” [76]. A typical example of such dysbiosis, characterized by a depletion of commensal bacteria such as *Bacteroidetes* and *Firmicutes* in contrast to an increase in *Proteobacteria* and *Actinobacteria,* has been identified as a key factor connected with the development of inflammatory bowel disease (IBD) [77]. Moreover, a specific genetic background can influence the host-microbial diversity (i.e., how bacterial species differ genetically, numerically and ecologically), richness and abundance [78]. For instance, variants of the human fucosyltransferase 2 (FUT2) gene affect the mucosal 1,2-fucosylated glycan structures (Fut-2), leading to a reduction in the *Bifidobacteria* population [79]. The same polymorphism is associated with CD susceptibility and IBD onset in the Finnish population [80]. The methods used to investigate the gut microbiota vary and include stool sampling, duodenal biopsies, the study of model organisms, culture systems, nucleotide sequencing (and related molecular biology techniques), transmission and scanning electron microscopy [81,82]; the choice of the method used and tissue sampling obviously affect the result. According to Bork et al., the gut microbiota has been divided into three enterotypes, also referred to as bacteriological ecosystems, each dominated by one of the three bacteria clusters: *Bacteroides* (enterotype I), *Prevotella* (enterotype II) and *Ruminococcus* (enterotype III) [75]. This classification is likely an oversimplification of microbiota structures, but it nonetheless points to a physiological implication since enterotypes show a different way to harvest energy [83]. Enterotype I bacteria are known to produce energy mainly from carbohydrates through glycolysis and pentose phosphate pathways, whereas enterotypes II and III degrade mucin glycoproteins of the gut mucosal layer. Dietary and cultural habits could influence the enterotypes and even finer grained species and strain-level microbial composition. Indeed, *Prevotella* species predominates among Africans having a generally low-fat and low-(animal) protein diet [84,85], whereas *Bacteroides*-rich microbiomes are more abundant among Europeans eating Western diet rich in lipids and animal proteins [72,75]. Although there are contrasting findings about the protective role of *Prevotella*, a genus comprising more than 40 species with a high genetic variability [86], many studies correlate the *Bacteroides* increase in the gut microbiota with the development of pathological conditions such as CD and IBD [87,88,89,90]. Deciphering the role of the different enterotypes is expected to provide informative data about gastrointestinal disorders.

## 6. Gut Microbiota Is Conditioned by Gluten in CD

Compared to asymptomatic controls, CD patients show a gut microbiota characterized by a higher number of total bacteria and a lower ratio of beneficial to harmful bacteria. These findings support the occurrence of gut dysbiosis in CD, which improves following gluten withdrawal [91,92]. A prototypic example of dysbiosis is given by small intestine bacterial overgrowth (SIBO), which may explain why some CD patients show poor responsiveness to GFD. Several studies have clearly indicated a beneficial effect on SIBO-related symptoms by using antibiotics, particularly the poorly absorbable class, such as rifaximin [93]. In CD, bacteria known for their protective effect, e.g., *Bifidobacteria*, *Firmicutes*, *Lactobacilli* and *Streptococceae*, are lower in number than in healthy controls, while the number of harmful Gram-negative bacteria (*Bacteroides*, *Bacterioidetes, Bacteroides fragilis*, *Prevotella*, *E. Coli*, *Proteobacteria, Haemophilus*, *Serratia*, *Klebsiella*) increase [94,95,96,97]. In duodenal biopsy of adult patients with active CD, *Proteobacteria* phylum and *Neisseria flavescens* were prominent, while *Firmicutes* and *Actinobacteria* were the least abundant [7,98]. These findings suggest that intestinal dysbiosis affects CD patients and contribute to persistent symptoms, even in those on a strict GFD regimen. An imbalance in the microbiota composition was also found by De Palma et al. in infants with genetic susceptibility to CD [99]. Indeed, feces of new-borns, bearing the HLA-DQ predisposing phenotype, were characterized by a higher number of *Bacterioides fragilis* and *Staphylococcus spp*. and a lower number of *Bifidobacteria* and *B. Longum* vs. healthy controls. The reason for this imbalance in the bacterial ratio could be ascribed to the glycocalyx mucous layer, a carbohydrate coating on the mucosal surface of the gastrointestinal tract. Each individual has a personal composition of the mucous glycocalyx (a genetically determined feature), which predisposes to CD by changing the specificity of bacterial adhesion and colonization. However, the hypothesis that the glycocalyx layer could play a role in CD onset has been recently questioned [100]. Breastfeeding is of crucial importance as it can help to restore the microbiota composition in babies carrying HLA-DQ2 haplotypes [99].

## 7. Gluten-Free Diet Effects on Healthy Human Microbiota

The overall literature search on databases including the terms “gluten free diet”, “GFD”, “gluten free diet AND healthy”, “microbiota”, “microbiome”, “microbiome AND healthy patients”, “microbiota AND healthy patients” produced 2775 results. Of these, excluding duplicates, three fulfilled our inclusion criteria. In 2009, De Palma et al. [101] explored whether a month of GFD affects the microbiota composition of ten healthy subjects. Enumeration of fecal bacteria by fluorescence in situ hybridization (FISH) using 16S rRNA-targeted oligonucleotide probes showed that GFD causes a decrease in the count of *Bifidobacterium*, *Clostridium lituseburense* and *Faecalibacterium prausnitzii*. Quantitative PCR (qPCR) characterization of fecal microbes following GFD revealed a reduction in the number of *Bifidobacterium*, Lactobacillus and *Bifidobacterium* longum and an increase in the *Enterobacteriaceae* and *Escherichia coli* counts. They propose that the depletion in *Bifidobacterium* and Lactobacillus, generally considered as probiotics, could be caused by the reduced availability of polysaccharides introduced with the GFD that serve as a substrate for gut microbiota. Moreover, the reduction in *Faecalibacterium prausnitzii*, along with the concomitant increase in the opportunistic pathogens *Enterobacteriaceae* and *Escherichia coli* in the fecal mucus of active Crohn’s disease patients was found to trigger the inflammatory insult [89,102,103]. Moreover, Hansen et al. showed that minimal amounts of gluten are sufficient to affect the microbiota population, lowering the *Bifidobacteria* count in patients adhering to a low-gluten regimen [104]. Indeed, the authors performed a randomized, controlled, cross-over trial study involving 60 non-CD Danish adults who followed a low-gluten diet (2 g gluten per day) for eight weeks and then switched to a high-gluten diet (18 g gluten per day) for another eight weeks, including a washout period of at least six weeks of normal diet (12 g gluten per day) between the two diets. Notably, GFD was associated with an increase of unclassified species of *Clostridiales* and an unclassified species of *Lachnospiraceae*, whereas *E. hallii* and *A. hadrus* (both butyrate-producers), *Dorea* (hydrogen producer) and the hydrogen-consumer and acetate-producer *Blautia*, in addition to two species of the *Lachnospiraceae* and four species of *Bifidobacterium*, were found to decrease. These microbial changes could be ascribed to the low-gluten diet availability of arabinoxylan and arabinoxylan-oligosaccharides, as these food components are abundant non-starch polysaccharides of cereal grains, which serve as energy substrates for the bacterial species mentioned above [105,106,107,108,109,110]. Bonder et al. [111] investigated the gut microbiota of 21 healthy volunteers on a GFD for four weeks, tested with a total of 9 stool samples for each person (one at baseline, four during the GFD and four when they returned to their usual diet). The microbiome profile was then characterized using 16 sRNA sequencing and investigated for taxonomic and implied functional compositions. Overall, the bacterial profile remained relatively stable in healthy individuals on GFD. However, some changes in the abundance of 8 families of bacteria were observed during the GFD period: *Veillonellaceae*, *Ruminococcus bromii* and *Roseburia faecis*, decreased, whereas *Victivallaceae*, *Clostridiaceae*, ML615J-28, *Slackia* and *Coriobacteriaceae* increased during GFD. *Veillonellaceae*, a pro-inflammatory family of Gram-negative bacteria known for lactate fermentation, increase in diseases such as IBD, irritable bowel syndrome and liver cirrhosis [88,112,113], while they decrease in autistic patients [114]. Compared to a normal diet, the abundance of *Ruminococcus bromii*, known to degrade the resistant starch in the human colon [115] and the cellulose, producing short chain fatty acids (SCFA) and hydrogen gas [116], was affected by the different starch composition of GFD. *Coriobacteriaceae* (*Slackia* genus in particular) and *Clostridiaceae* were associated with CD, IBD and colorectal cancer [117,118,119]. Thus, gluten withdrawal alters mostly bacterial species, utilizing carbohydrate and starch as energy substrates.

**Figure 1:**
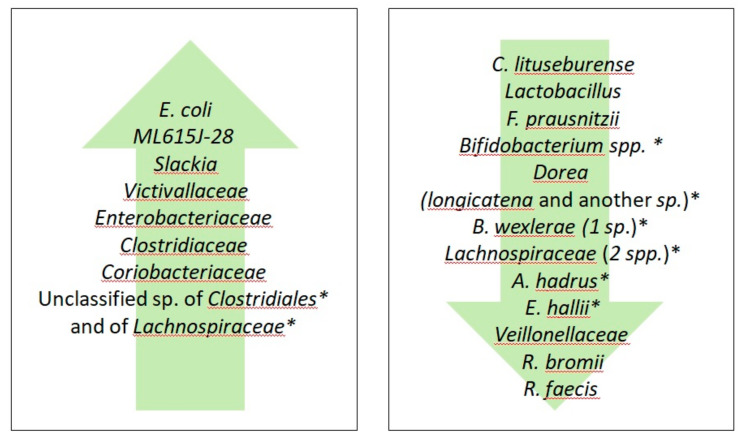
Effects of gluten-free diet (GFD) on the abundance of the bacterial populations in healthy patients. * In a low-gluten containing diet.

The effects of GFD on the abundance of bacterial populations in healthy patients are illustrated in Figure 1.

## 8. Gluten-Free Diet Effects on Microbiota of CD Patients

Although many studies have investigated the effects of GFD on the gut microbiota of healthy people, data on the change of the microbial community induced by GFD in adult CD patients are very limited. The overall literature search on databases including the terms “microbiome AND celiac disease”, “microbiome AND gluten free diet”, “microbiota AND celiac disease”, “microbiota AND gluten free diet”, “gluten free diet AND adult”, “celiac disease AND adult” produced 3780 results. Excluding duplicates, six publications were selected as the most relevant for the purposes of this review and detailed below. Golfetto et al. [120] examined the feces of 42 healthy subjects and 14 celiac patients in GFD for at least two years. The concentration of fecal *Bifidobacteria* and fecal pH were determined for both groups in order to establish if two years of GFD were sufficient to restore *Bifidobacteria*, which are usually scarce in CD patients compared to healthy subjects. The results showed that CD patients with no active disease had less concentration of *Bifidobacteria* per gram of feces vs. healthy subjects (2.5 ± 1.5 × 10^7^ CFU/g of celiac patients against 1.5 ± 0.63 × 10^8^ CFU/g of healthy subjects), whereas no differences were found in terms of fecal pH between the two groups. These findings suggested that an imbalance in the intestinal microbiota of CD patients, due to a reduced *Bifidobacteria* population, may be a co-factor triggering the disease. Moreover, a *Bifidobacteria* depletion was detected in duodenum as well as in the feces of CD and atopic children [91,121].

Wacklin et al. [122] compared the duodenal microbiota profile of GFD-treated CD patients with and without persistent symptoms and found that the GFD-treated symptomatic group (i.e., non-responsive CD patients) showed an overall reduction in bacterial richness. Compared to GFD-treated asymptomatic patients, the microbiota of GFD-treated CD patients with persistent symptoms displayed an abundance of *Proteobacteria* and low amounts of *Bacteroidetes* and *Firmicutes*. Nistal et al. [123] analyzed feces of CD patients under GFD for at least 2 years (treated CD, TCD), compared with CD patients following a normal gluten dietary regimen (untreated CD, UCD) and healthy controls. Stools of TCD were characterized by a reduced diversity of *Lactobacillus* and *Bifidobacterium* species, whereas *Bifidobacterium bifidum* in UCD was higher vs. controls. Moreover, analysis of SFCAs revealed that the majority of TCD had a fecal microbiota composition similar to healthy controls, indicating that GFD could normalize gut microbiota composition in CD. In another study, Nistal et al. focused on the microbiota richness of the upper small intestine mucosa of adult TCD, UCD and healthy subjects [124]. They used 16S RNA gene sequencing from duodenal biopsies to identify intestinal bacterial communities and Unifrac online servers to determine the phylogenetic distance between bacterial communities. The study concluded that TCD have very different bacterial communities from UCD and that GFD decreased bacterial richness in TCD (17 genera) vs. UCD (24 genera) or healthy controls (21 genera). Among these genera, *Streptococcus* and *Prevotella* were predominant, while seven unknown genera were found only in UCD and healthy controls. Moreover, *Prevotella* and *Streptococcus* were reduced in UCD, while they remain similar in number in TCD and healthy controls. Interestingly, only healthy people had their upper part of the small intestine populated by *Streptococcus* and *Haemophilus*, which are microorganisms typically found in the oro-pharyngeal tract [125]. This was a very surprising finding, as the upper small intestine (low pH, peristalsis, mix of nutrients with secretions, bile and proteolytic enzymes) is expected to prevent any microbial survival [126]. Moreover, it was found that a correlation between the colorectal cancer and the presence of oral cavity-associated species in the gut, which are conversely very low in number in controls [127].

A study by Caminero et al. [128] investigated microbial community composition and gluten metabolism in CD patients in GFD for at least one year, UCD, CD relatives and healthy controls. Data obtained analyzing fecal samples of UCD showed very low concentrations of *Lactobacillus* and a significant increase of *Clostridium* vs. healthy volunteers and CD relatives. The bacterial metabolism was investigated by analyzing the fecal concentration of SCFA (e.g., acetic, propionic and butyric acid) and fecal tryptic activity. UCD subjects and their first degree relatives showed a higher concentration of SCFA compared with healthy controls, while CD patients had a higher fecal tryptic activity compared to healthy volunteers and first-degree relatives on either normal diet or GFD. SCFA branches were lower in CD on GFD for one year than in their first-degree relatives following GFD for only a month, suggesting that a prolonged GFD could modify SCFA proteolytic patterns. They also found constantly altered fecal glutenous activity in all CD subjects, even in GFD-treated patients with a fully restored mucosa (i.e., villi regrowth). The altered fecal glutenous activity was associated with an unbalanced gut microbiota function, as indicated by high microbiota-related endo-prolyl peptidases activity (gliadinase) found in duodenal samples of GFD or non-GFD-treated CD patients, but not in healthy subjects [129]. Thus, dysbiosis may affect the metabolism of gluten proteins in CD patients as a result of an imbalance in the commensal microbiota composition.

D’Argenio et al. [98] examined the microbiota composition of duodenal samples of 20 patients with active CD on a normal gluten-containing diet, of 6 patients adhering to GFD for at least 2 years and 15 control patients. The 16S bacterial rRNA sequencing of duodenal biopsies revealed 20 different phyla, the most abundant ones being *Proteobacteria* (most abundant phylum in all the investigated groups), *Actinobacteria, Bacteroidetes, Firmicutes* and *Fusobacteria*. Compared to CD patients in GFD and controls, duodenal biopsies of active CD patients revealed a higher abundance of the *Betaproteobacteria* class, belonging to *Proteobacteria* phylum, and a lesser abundance of the *Gammaproteobacteria* class, along with a reduced presence of *Actinobacteria* and *Firmicutes*. Finally, Bodkhe et al. [130] examined differences in the composition of the gut microbiome of healthy controls, CD patients and their first-degree relatives, who are genetically susceptible to CD, and therefore a good model of the pre-disease state before patients started GFD. The duodenal microbiota of first-degree relatives showed a greater abundance of *Parvimonas*, *Granulicatella, Gemella*, *Bifidobacterium, Anaerostipes* and *Actinomyces* genera compared to controls and CD subjects, the latter group being characterized by an abundance of *Megasphaera* and *Helicobacter* genera. Moreover, stool samples of CD and first-degree relatives displayed less abundance of *Akkermansia* and *Dorea* compared to healthy controls, whereas CD subjects showed a reduced gluten degradation ability compared to first-degree relatives and controls.

**Figure 2:**
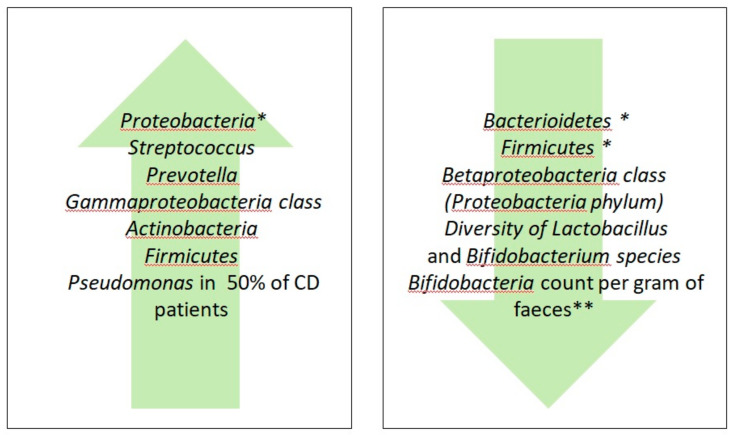
Effects of gluten-free diet (GFD) on the abundance of bacterial populations in Celiac disease (CD) patients. * In CD patients with persisting gastrointestinal symptoms; ** In CD patients with no active disease.

The effects of GFD on the abundance of bacterial populations in CD patients are illustrated in Figure 2.

## 9. GFD Effects on Microbiota of NCG/WS Patients

Gluten, alone or in combination with FODMAPS, has been considered one of the major triggers of NCG/WS. Gluten reduction/withdrawal from the diet is accompanied by significant symptom improvement in affected patients [41,45,57]. Despite many progresses in a better understanding of the importance of GFD in GRDs, the effect of gluten withdrawal on the gut microbiota of NCG/WS patients is a challenging topic, which so far remains poorly elucidated. The overall literature search on databases including the terms “microbiome AND non celiac gluten sensitivity”, “microbiome AND non celiac wheat sensitivity”, “microbiota AND non celiac gluten sensitivity”, “microbiota AND non-celiac wheat sensitivity”, “non-celiac wheat sensitivity AND adult”, “non-celiac gluten sensitivity AND adult”, “gluten free diet AND leaky gut”, “non-celiac gluten sensitivity AND leaky gut”, “non-celiac wheat sensitivity AND leaky gut” produced more than 103 results. Excluding duplicates, three publications appraised the impact of GFD on gut microbiota changes in patients with “non-celiac gluten sensitivity” and have been discussed in this section.

Mazcorro et al. [131] assessed the gut microbiota of Mexican patients affected by CD (*n* = 6), NCG/WS (*n* = 12) and healthy subjects (*n* = 12). Both the duodenal and the fecal microbiota of patients and controls were analyzed by ultra-high-throughput 16S rRNA gene marker sequencing. The same investigation was repeated after four weeks of GFD, considering a subgroup of patients with available paired samples. As a whole, each of the three groups under investigation showed changes of the gut microbiota following the GFD period, suggesting that gluten withdrawal can significantly affect microbial composition. *Actinobacillus* genus and the *Ruminococcaceae* family were predominant in the duodenal and fecal microbiota of NCG/WS patients, whereas *Novispirillum* was more abundant in the duodenum of CD patients. Moreover, in the feces of CD patients was found a higher amount of *Veillonellaceae*, a pro-inflammatory taxon reported to increase in IBD [88,112,113]. Paired samples from NCG/WS patients showed a significant difference in duodenal *Pseudomonas* concentration vs. the baseline period. Some CD patients (4/6) and almost all NCGS patients (9/10) following GFD for four weeks reported an improvement in symptoms and quality of life, suggesting that this period of time was sufficient to change the gut microbiota in each group. However, for a complete restoration of gut microbiota function, a longer period of GFD was recommended [132]. Surprisingly, both 9 out 10 of fecal paired samples of NCG/WS subjects and half of the fecal paired samples from the CD individuals (3/6) showed an unexpected rise in the *Pseudomonas* concentration after the GFD period. The genus *Pseudomonas* is highly heterogenic and, although it has been often associated with diseases, there are thousands of non-pathogenic strains [133]. Two hypotheses have been proposed by the authors to explain this unusual finding. First, the low concentration of *Pseudomonas* in the mucosa at the baseline may be due to the immunological status in some patients with NCG/WS. Secondly, *Pseudomonas* could act as a protective agent and its reduced presence in CD and NCG/WS patients at the baseline could favor sensitivity to dietary allergens. These findings open up a new role for *Pseudomonas* as a protective agent and even as a probiotic for CD and NCG/WS patients. A protective role of *Pseudomonas* for gut mucosa has already been proposed by Gao et al., [134] showing that these taxa decrease in cancer tissue compared to controls. Wei et al. [135] found that *P. aeruginosa* produces the gluten-degrading enzyme pseudolysin, exhibiting a possible therapeutic potential for CD. This finding supported D’Argenio et al., who found an abundance of *Pseudomonas* in the duodenum of adult CD patients on a GFD compared to controls [98]. Other non*-Pseudomonas Proteobacteria* (e.g., *Stenophomonas*) that could play a role in gluten degradation and distal gut microbiota deserves attention as a potential factor able to influence various GRDs.

Dieterich et al. [60] combined the GFD with low FODMAP diet in patients with NCG/WS. Enrolled patients consumed a gluten-containing standard diet before starting a two-week low FODMAP diet; then, after a five-day transition period, they adhered to a GFD for another two weeks. NCG/WS patients reported an improvement of the overall wellbeing under different diets in association with a reduction of intestinal inflammation. Both diets caused a huge impact on the microbiota population of patients with NCG/WS, with the low FODMAP diet being associated with an increased *Lachnospiraceae* and diminished *Bifidobacteriaceae*, while the GFD increased *Bacteroidaceae,* but decreased *Lachnospiraceae*. Moreover, in NCGS patients, there was a reduction of duodenal intraepithelial lymphocytes and mucin-producing goblet cells, whereas bacterial variability increased. Moreover, it was found that the gut microbiota abnormalities affect NCG/WS patients’ metabolism.

## 10. A Gluten-Free Life: Agri-Food Technologies

**Figure 3:**
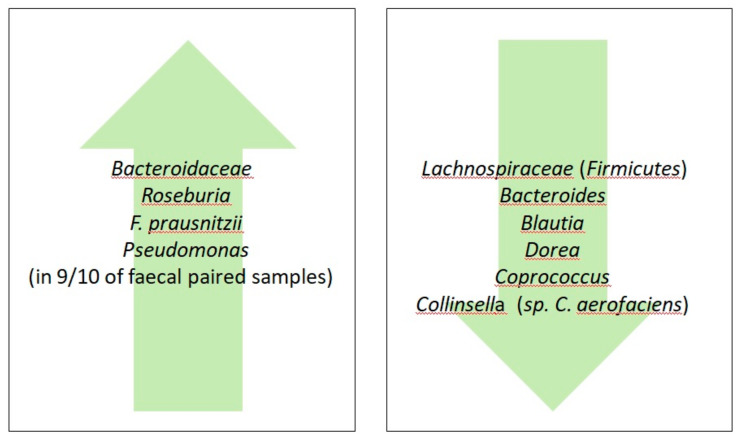
Effects of GFD on the abundance of bacterial populations in non-celiac gluten/wheat sensitivity (NCG/WS) patients.

In a world witnessing an increasing prevalence of GRDs [136,137], industries aim to satisfy the demand of gluten-free food by designing and producing new technology-driven ad hoc products. Accordingly, Haro et al. evaluated the effects of transgenic low gliadins wheat bread on intestinal microbiota composition in patients affected by NCG/WS [138]. Transgenic wheat was characterized by very low gliadins (90% less than the wild type wheat), reduced low molecular weight glutenins and high non-gluten protein content. The study was designed in two parts: (*i*) patients followed a strict GFD (basal period); (*ii*) patients switched from gluten-free to a transgenically modified wheat bread (second period). At the end of each phase (lasting seven days), fecal samples were collected, and the bacterial 16S rRNA gene V1-V2 hypervariable regions of fecal samples were amplified by PCR, sequenced and clustered. In stools of phase two, there was an increase in *Roseburia* and *Faecalibacterium* genera, known to play an anti-inflammatory role for their butyrate production, enhancing TJ expression and intestinal barrier integrity. In contrast, *Bacteroides* (pro-inflammatory), *Blautia*, *Dorea*, *Coprococcus* and *Collinsella* (species *C. aerofaciens*) decreased, suggesting that low gliadins bread could lower inflammation and improve gut permeability, while maintaining the same non-gluten protein content of wild type bread. The effects of GFD on the abundance of bacterial populations in NCG/WS patients are illustrated in Figure 3.

The main studies quoted in this review regarding the effects of GFD on the microbiota of healthy subjects and CD and NCGS patients are summarized in Table 1 and Table 2.

## 11. Conclusions

Growing evidence indicates that the interplay between gut microbiota and intestinal epithelial barrier function play a critical role in priming and maintaining a competent immune system. All together, these factors generate a gastrointestinal ecosystem, which, in concert with the classic repertoire of gut physiology, prevent the detrimental effect of various *noxae*. Offending foods belongs to those harmful substances able to perturb the gastrointestinal ecosystem, thereby leading to disease states. In this wide research area that is still far from being clarified, even classic dietary factors, such as wheat and related gluten and amylase trypsin inhibitors, can play a role in symptom generation in genetically susceptible or sensitive patients.

This review appraised the current knowledge about the gut microbiota in health as well as CD and NCG/WS and the related effects evoked by GFD in these two most common conditions. The evidence so far acquired has demonstrated that diseases are often characterized by an imbalance in the microbial intestinal population composition, leading to dysbiosis, a condition promoting inflammation and metabolic impairment. In CD, the depletion of probiotic species, i.e., Lactobacillus and Bifidobacteria and the relative increase of pro-inflammatory bacteria, e.g., Veillonaceae genus, represent microbiota fingerprints likely contributing to disease onset, which is common to CD patients. In all the groups analyzed, GFD was shown to reduce bacterial richness while affecting gut microbiota composition in a different manner depending on health (asymptomatic subjects) and disease state (CD and NCG/WS). Indeed, in healthy subjects, GFD causes the depletion of beneficial species, e.g., Bifidobacteria, in favour of opportunistic pathogens, e.g., Enterobacteriaceae and Escherichia coli. Conversely, in CD and NCG/WS, GFD evoked a positive effect on gastrointestinal symptoms by helping to restore the microbiota population and by lowering pro-inflammatory species.

In conclusion, these studies shed light on the complex interactions occurring between diet, gut barrier and gut microbiota. Multiple aspects are still to be explored along the microbiome-diet axis, including investigations into the yet-to-be-defined species that constitute large fractions of the microbiome [84], as well as the role of strain-specific microbial determinants and the difficulties in capturing detailed dietary information in large diverse metagenomics cohorts. In addition to general investigations of the complex link between diet, microbiome and health, further studies are particularly needed to specifically improve our knowledge of the effects that GFD could exert on the bacterial species involved within CD and NCG/WS.

## Figures and Tables

**Table 1:** Main findings of the studies evaluating the effect of gluten-free diet (GFD) on the gut microbiota in healthy subjects and in patients with celiac disease (CD) and non-celiac gluten/wheat sensitivity (NCG/WS). All investigated patients are adults with the exception of Nistal et al., 2012 [124] in which children have been also investigated.

	Author, Year and Reference Number	Subjects Investigated *	GFD Duration	Sample/Methods	Main Findings
Healthy Subjects	De Palma et al., 2009 [101]	healthy subjects (*n* = 10)	1 month	Feces; FISH and qPCR	↓ Bifidobacterium, Bifidobacterium longum, Lactobacillus, Clostridium lituseburense, Faecalibacterium prausnitzii count; ↑ Enterobacteriaceae and Escherichia coli
	Hansen et al., 2018 [104]	non-CD subjects (*n* = 60)	low gluten diet (2 g of gluten/day) for 8 weeks; then a washout period of at least 6 weeks of normal diet (12 g of gluten/day) followed by 8 weeks of high-gluten diet (18 g of gluten/day)	Feces; Metagenomic sequencing and qPCR	↓ Bifidobacterium spp., Dorea longicatena and another species of Dorea, 1 species of Blautia wexlerae, 2 species of the Lachnospiraceae family and Anaeostipes hadrus and Eubacterium hallii; ↑ unclassified species of Clostridiales and an unclassified species of Lachnospiraceae
	Bonder et al., 2016 [111]	healthy subjects (*n* = 21)	4 weeks	Feces; 16S rRNA sequencing	Bacterial flora remains stable, even on GFD; ↓ Veillonellaceae, Ruminococcus bromii and Roseburia faecis; ↑ Victivallaceae, Clostridiaceae, ML615J-28, Slackia and Coriobacteriaceae
CD	Golfetto et al., 2014 [120]	healthy subjects (*n* = 42) and CD patients (*n* = 14)	2 years	Feces; CFU/gram of fecal sample weight. Gram stain, catalase test and fructose-6-phosphate phosphoketolase test	↓ Bifidobacteria count/gram of faeces in CD patients with no active disease vs. healthy subjects
	Wacklin et al., 2014 [122]	CD patients in GFD with (*n* = 18) and without (*n* = 18) persisting gastrointestinal symptoms	≥3 years	Duodenal biopsies; 16S rRNA gene pyrosequencing	CD patients with persistent symptoms on GFD had altered duodenal microbiota composition and ↓ microbiota richness vs. CD patients without symptoms; ↑ Proteobacteria in patients with persisting gastrointestinal symptoms; ↓ Bacterioidetes and Firmicutes in patients with persisting gastrointestinal symptoms
	Nistal et al., 2012 [123]	Untreated CD patients (i.e., on gluten-containing diet) (*n* = 10); GFD treated CD patients (*n* = 11) and healthy subjects on gluten-containing diet and GFD for 1 week (*n* = 11)	At least 2 years	Feces; DGGE and gas-liquid chromatography for SCFAs	↓ Lactobacillus and Bifidobacterium species diversity of GDF treated CD patients; ↑ Bifidobacterium bifidum in GFD untreated CD patients vs. healthy subjects; GFD treated CD patients have similar levels of SCFA vs. healthy subjects
	Nistal et al., 2012 [124]	GFD treated adult CD patients (n = 5), GFD untreated CD patients (on gluten-containing diet) (*n* = 5) and healthy subjects (*n* = 5);GFD untreated CD children (*n* = 8) and healthy children (*n* = 5)	Not specified	Duodenal biopsies; 16S rRNA gene sequencing	GFD treated CD (TCD) show very different bacterial communities from GFD untreated CD (UCD) patients; ↓ bacterial richness in TCD; Streptococcus and Prevotella predominate in TCD; 7 unknown genera found only in UCD and healthy controls; ↓ Prevotella and Streptococcus in UCD; Streptococcus and Haemophilus populated the upper part of the small intestine of healthy subjects, but not CD
	Caminero et al., 2015 [128]	UCD (*n* = 22), TCD (*n* = 20), CD relatives on normal diet (*n* = 71) and on GFD (*n* = 69), healthy subjects on normal diet (*n* = 16) and on GFD (*n* = 11)	CD patients on GFD for at least 1 year; relatives on a GFD for 1 month;Healthy subjects on GFD for 1 week	Feces; 16S rDNA gene partial sequencing	↓ Lactobacillus in feces of UCD patients; ↑ Clostridium in feces of UCD patients; ↑ SCFA in UCD and their relatives; ↑ FTA in CD patients; ↓ SCFA branches in CD patients on GFD for 1 year; Altered fecal glutenous activity in all CD patients
	D’Argenio et al., 2016 [98]	UCD (*n* = 20), TCD (*n* = 6) for at least 2 years and control subjects (*n* = 15)	At least 2 years	Duodenal biopsies; 16S rRNA sequencing	20 different phyla; The most abundant phyla were Proteobacteria, Actinobacteria, Bacteroidetes, Firmicutes (same amount in all groups) and Fusobacteria; ↑ Betaproteobacteria class belonging to Proteobacteria phylum in UCD patients; ↓ Gammaproteobacteria class, Actinobacteria and Firmicutes in UCD patients
NCG/WS	Mazcorro et al., 2018 [131]	CD (*n* = 6), NCG/WS (*n* = 12) and healthy subjects (*n* = 12)	4 weeks	Feces; duodenal biopsy;16S rRNA gene sequencing	Actinobacillus genus and the family Ruminococcaceae predominate in the duodenal and fecal microbiota of NCG/WS patients; ↑ Novispirillum in the duodenum of CD patients; ↑ Veillonellaceae in fecal samples of CD patients; ↑ Pseudomonas in 9 out of 10 fecal paired samples of NCG/WS patients on GFD; ↑ Pseudomonas in only 50% of fecal paired samples from TCD patients
	Dieterich et al., 2019 [60]	NCG/WS patients (*n* = 19) healthy controls (*n* = 10)	standard gluten-containing diet before starting a 2-week of low FODMAP diet; 5-day transition period, then GFD for another 2 weeks	Feces; 16S rRNA gene sequencing	↑ Bacteroidaceae in GFD treated NCG/WS patients; ↓ Lachnospiraceae (Firmicutes); ↑ low FODMAP diet Lachnospiraceae (Firmicutes); ↓ Bifidobacteriaceae (Actinobacteria)
	Haro et al., 2018 [138]	NCG/WS patients (*n* = 10)	7 days of GFD (basal period); then 7 days of GFD with the substitution of the gluten-free diet with the low-gliadin bread (second period)	Feces; 16S rRNA gene sequencing	↑ Roseburia and Faecalibacterium prausnitzii in feces collected during the second period; ↓ Bacteroides, Blautia, Dorea, Coprococcus and Collinsella (species C. Aerofaciens)

* CFU: colony forming units; DGGE: denaturing gradient gel electrophoresis; FDR: first degree relatives; FISH: fluorescence in situ hybridization; FTA: fecal tryptic activity; qPCR: quantitative polymerase chain reaction; SCFA: short chain fatty acids; TCD: GFD treated CD; UCD: GFD untreated CD. FODMAP: fermentable oligosaccharides, disaccharides, monosaccharides and polyols. ↑ means the increasing of bacterial populations following GFD, ↑ means the decreasing of bacterial populations following GFD.

**Table 2:** Effects of GFD of low-gluten diet on gut microbiota of healthy subjects compared to CD and NCG/WS patients.

Main Effects of GFD on Gut Microbiota of Healthy Subjects	Main Effects of GFD on Gut Microbiota of Celiac Disease	Main Effect of GFD on Gut Microbiota of Non-Celiac Gluten/Wheat Sensitivity
↑ Enterobacteriaceae and Escherichia coli [101] ↑ unclassified species of unknown taxonomic origin, an unclassified species of Clostridiales and an unclassified species of Lachnospiraceae [104] * ↑ Victivallaceae, Clostridiaceae, ML615J-28, Slackia and Coriobacteriaceae [111] ↓ Bifidobacterium, Lactobacillus, Clostridium lituseburense, Faecalibacterium prausnitzii [101] ↓ Bifidobacterium spp., Dorea longicatena and another species of Dorea, one species of Blautia wexlerae, two species of the Lachnospiraceae family, Anaeostipes hadrus and Eubacterium hallii [104] * ↓ Veillonellaceae, Ruminococcus bromii and Roseburia faecis [111]	↑ *Proteobacteria* and ↓ *Bacterioidetes* and *Firmicutes* in CD patients with persisting gastrointestinal symptoms [122] ↑ *Streptococcus* and *Prevotella* in TCD, whereas ↓ in UCD [124] ↑ *Pseudomonas* in 50% of TCD [131] ↓ *Bifidobacteria* count per gram of feces in CD patients with no active disease [120] ↓ Bacteria richness [124] ↓ Bacterial diversity of *Lactobacillus* and *Bifidobacterium* (*Bifidobacterium bifidum*) [123]	↑ *Pseudomonas* in 9 out of 10 fecal paired samples of patients after 4 weeks of GFD [131] ↑ *Bacteroidaceae* [60] ↑ *Roseburia* and *Faecalibacterium (species prausnitzii*) [138] ↓ *Lachnospiraceae* (*Firmicutes*) [60] ↓ *Bacteroides*, *Blautia*, *Dorea*, *Coprococcus* and *Collinsell*a (species *C. Aerofaciens*) [138]

CD: celiac disease; GFD: gluten-free diet; NCG/WS: non-celiac gluten/wheat sensitivity; TCD: GFD treated CD; UCD: GFD untreated CD. * in a low gluten containing diet. ↑ means the increasing of bacterial populations following GFD, ↑ means the decreasing of bacterial populations following GFD.
